# Association of smoking with myocardial injury and clinical outcome in patients undergoing mechanical reperfusion for ST-elevation myocardial infarction

**DOI:** 10.1186/1532-429X-18-S1-Q19

**Published:** 2016-01-27

**Authors:** Ingo Eitel, Sebastian J Reinstadler, Charlotte Eitel, Suzanne de Waha, Georg Fuernau, Steffen Desch, Holger Thiele

**Affiliations:** Cardiology, Angiology, Intensive Care Medicine, University Heart Center Lübeck, Lübeck, Germany

## Background

There is evidence suggesting a positive effect of cigarette smoking on myocardial tissue reperfusion and clinical outcomes in patients with myocardial infarction ("smoker's paradox"). We aimed to evaluate the relationship of smoking status with cardiac magnetic resonance (CMR)-determined myocardial salvage and damage as well as clinical outcome in patients undergoing primary percutaneous coronary intervention (PPCI) for ST-elevation myocardial infarction (STEMI).

## Methods

This multicenter study included 727 consecutive STEMI patients reperfused within 12 hours after symptom onset. CMR imaging parameters (area-at-risk [AAR], infarct size [IS], myocardial salvage index [MSI], and microvascular obstruction [MVO]) were compared according to admission smoking status. Major adverse cardiac events (MACE) rates at 12 months after infarction were compared between groups.

## Results

In our study cohort 339 (46.6%) patients were current smokers. There was no difference in the extent of AAR (35[24-47] vs. 37[27-49] %LV, p = 0.10), IS (16[8-25] vs. 17[10-26] %LV, p = 0.21), MSI (53[29-70] vs. 52[34-71], p = 0.47) or MVO (0[0-1.7] vs. 0[0-1.6] %LV, p = 0.91) between smokers and non-smokers. Smokers had lower MACE (3.8% vs. 8.2%, p = 0.01) and mortality (0.9% vs. 3.9%, p = 0.01) rates. However, after adjustment for differences in baseline risk factors, smoking was no longer associated with MACE (HR = 0.72, 95% CI 0.37 to 1.41, p = 0.34) or mortality (HR = 0.49, 95% CI 0.14 to 1.76, p = 0.27).

## Conclusions

Smoking is not associated with PPCI efficacy (myocardial salvage) or irreversible myocardial damage in patients with acute STEMI. The lower MACE and mortality rates of smokers were entirely explained by differences in baseline risk characteristics, thus challenging the existence of a "smoker's paradox".

